# Editorial: The Oxytocin System in Fear, Stress, Anguish, and Pain

**DOI:** 10.3389/fendo.2021.737953

**Published:** 2021-07-20

**Authors:** Adam S. Smith, Benjamin Jurek, Valery Grinevich, Michael T. Bowen

**Affiliations:** ^1^ Department of Pharmacology & Toxicology, University of Kansas, Lawrence, KS, United States; ^2^ Institute of Molecular and Cellular Anatomy, University of Regensburg, Regensburg, Germany; ^3^ Department of Neuropeptide Research in Psychiatry, Central Institute of Mental Health, Medical Faculty Mannheim, University of Heidelberg, Mannheim, Germany; ^4^ Brain and Mind Centre, University of Sydney, Sydney, NSW, Australia; ^5^ Faculty of Science, School of Psychology, University of Sydney, Sydney, NSW, Australia

**Keywords:** oxytocin, fear, anxiety, trauma, sex differences, pain

For over a decade the hypothalamic neuropeptide and peripheral neurohormone oxytocin (OT) has attracted considerable attention from both the scientific and broader community for its apparent “pro-social” effects: increasing the salience of social cues and facilitating social communication, interaction, trust, openness, generosity and empathy. However, it is becoming increasingly apparent that OT has a variety of functions beyond its centrally mediated effects on social behavior and peripherally mediated reproductive functions.

Emerging evidence supports a role for OT in fear, stress, anguish and pain. Moreover, the involvement of OT in these phenomena is at least partly subserved by interactions with distinct neural circuits from those involved in the pro-social effects of OT. This Research Topic focuses on these areas of growing interest in OT research, and how OT’s social and non-social effects can have bidirectional interactions. Areas covered include: research showing activation of the OT system in a rodent model of mono-arthritis; an exploration of mechanisms driving OT’s seemingly contradictory anxiolytic and anxiogenic effects; clinical research demonstrating how stress and trauma can undermine the capacity of the OT system to support the default mode network; the complex effects of OT on memory processes, fear and depression-like behavior in high-fat-diet-fed mice; the role OT plays in regulating the salience of fear-related stimuli and contexts; OT and vasopressin (AVP) involvement in sex differences in the dysregulation of emotional behavior in mandarin voles following chronic social stress; and a study exploring how OT might regulate anxiety, at least in some contexts, by facilitating attentional bias toward the eyes on familiar faces.

Acute nociceptive stimuli evoke multiple neuroendocrine responses, including activation of the HPA axis and several neuropeptide systems [i.e., OT, AVP, and corticotropin-releasing hormone (CRH)]. Using an acute mono-arthritic model in adult male Wistar rats, Nishimura et al. documented increased activity and expression of OT and AVP neurons in the paraventricular nucleus of the hypothalamus, increased CRH expression in the anterior pituitary, and elevated plasma levels of OT, AVP, and corticosterone. These results identify the activation state of OT and AVP systems and the HPA axis during acute mono-arthritis.

OT is associated with both anxiolytic and anxiogenic effects. Jurek and Meyer review literature which supports the modulatory role of OT in the social salience network, which evokes context-dependent behavioral outcomes, including anxiolysis and anxiogenesis. Further, they hypothesize that MEF2 is a key element which modulates the connectivity of OT receptor-expressing neurons, giving rise to this behavioral variability.

Genetic variability of the OT receptor can alter stress responsivity and stress-related psychopathology through modulation of neural activity and connectivity. Zeev-Wolf et al. examined the association between OT receptor gene variance and default mode network connectivity at the transition to adolescence and how childhood trauma impacts this relationship. Specifically, multiple single nucleotide polymorphisms of the OT receptor gene, trauma exposure, and avoidant symptoms in childhood combined to explain much of the variance in the connectivity of the default mode network. This suggests that OT supports default mode network maturation, but early life stress and psychological manifestations of stress undermine its function.

OT influences social salience and social information processing. This has been demonstrated in a number of paradigms, including eye gazing. Marsh et al. investigated whether the influence of OT on eye gazing is dependent on the familiarity of faces. Intranasal administration of OT increased eye gazing compared to placebo but only when the face was familiar (i.e., a partner or close friend). Furthermore, individuals who scored high on autistic-like traits had reduced eye gazing regardless of familiarity and OT treatment. Thus, OT facilitates an attentional bias towards eye gazing with familiar faces, which may convey safety and support.

The endogenous OT system plays an important role in the regulation of food intake and subsequent psychological consequences. In the study by Hayashi et al., i.p. OT administration to obese male mice improved social recognition, object-recognition and depressive-like behavior, but increased anxiety-like behavior. OT mRNA and protein expression were increased in the hypothalamus after OT treatment. In contrast, OT receptor mRNA was downregulated in the hippocampus, with or without OT treatment, identifying a possible neural mechanism involved in obesity-induced memory impairments. In summary, i.p. OT has some beneficial effects on recognition memory processes; however, as indicated by previous studies, it can have adverse effects on anxiety.

The OT system’s involvement in fear, anxiety and stress-coping reveals interesting sex differences on a cellular and behavioral level. The study by Hou et al. investigates chronic social defeat stress in male and female mandarin voles with respect to social and stress-coping behaviors. Both sexes react to chronic social defeat with social avoidance. However, the OT system of female mandarin voles seems to be more sensitive, as evidenced by reduced c-Fos expression in OT cells in the PVN, paralleled by reduced locomotor activity and negative coping behaviors. In contrast, males only show social avoidance and increased activation of the vasopressin system. These results highlight the importance of considering sex and neuropeptide systems when assessing resilience or vulnerability to social stressors.

The “social salience hypothesis” posits that OT regulates attention to social cues. Specifically, OT acts to increase attention to both negative and positive social cues. This hypothesis reconciles effects of oxytocin which were, under previous theories, seeming contradictory. The review by Olivera-Pasilio and Dabrowska extends the social salience hypothesis by proposing that OT also enhances the salience of non-social cues in non-social contexts. To support this, they review literature demonstrating the complex effects of OT on fear responses. For instance, OT facilitates active escape behavior to imminent threats, but reduces fear towards diffuse, non-imminent threats. Projections between the basolateral amygdala, the BNST, PVN/SON/AN, and the central amygdala appear to be involved in OT’s regulation of fear responses. The evidence presented thus demonstrates the important role OT plays in augmenting the salience of social and non-social cues.

Overall, the work presented in this Research Topic demonstrates that characterizing OT as “the social neuropeptide” belies the complexity of its effects. It is clear that OT not only has a powerful regulatory role in social behavior, but also on processes involved in fear, stress, anguish and pain. Moreover, bidirectional interactions are likely involved in at least some of OT’s effects across these domains.

## Author Contributions

All authors contributed to the article and approved the submitted version.

## Conflict of Interest

MB is listed as an inventor on patents for novel oxytocin-based therapeutic candidates and is co-founder and chief scientific officer of a company, Kinoxis Therapeutics Pty Ltd, commercializing some of this technology.

The remaining authors declare that the research was conducted in the absence of any commercial or financial relationships that could be construed as a potential conflict of interest.

